# *Streptococcus pyogenes* bacteremia and toxic shock syndrome related to *Strongyloides stercoralis* hyperinfection: a case report

**DOI:** 10.1186/s13256-018-1885-7

**Published:** 2018-11-22

**Authors:** Regev Cohen, Talya Finn, Frida Babushkin, Maurice Shapiro, Martina Uda, Tamar Grossman

**Affiliations:** 1 Infectious Diseases Unit, Sanz Medical Center, Laniado Hospital, Netanya, Israel; 2 Ruth and Bruce Rappaport Faculty of Medicine, Technion, Haifa, Israel; 3 Intensive Care Unit, Sanz Medical Center, Laniado Hospital, Netanya, Israel; 4 Reference Parasitology Laboratory, Central Laboratories, Public Health Services, Ministry of Health, Jerusalem, Israel

**Keywords:** *Strongyloides stercoralis*, *Streptococcus pyogenes*, Group A streptococcus (GAS), Streptococcal toxic shock syndrome (STSS), Hyperinfection, Eosinophilia, Glucocorticoid

## Abstract

**Background:**

We describe a patient with *Strongyloides stercoralis* hyperinfection associated with *Streptococcus pyogenes* and with streptococcal toxic shock syndrome. To the best of our knowledge this association has not been previously described.

**Case presentation:**

A 78 year-old Israeli man, who was born in Iraq but lived in Israel for 66 years, presented with multi-organ failure including acute kidney and hepatic injury, coagulopathy, and lactic acidosis. He had a medical history including aortic valve replacement, diabetes mellitus, spinal stenosis, and low back pain treated with repeated local steroid injections. Blood cultures were positive for *Streptococcus pyogenes* and antibiotic treatment was switched to penicillin G, clindamycin, and intravenous immunoglobulins. Repeated physical examinations failed to identify the source of the bacteremia. On day 12 of hospitalization the serology results for *Strongyloides stercoralis* sent on admission, because of chronic eosinophilia, came back positive. A microscopic stool examination and stool polymerase chain reaction were positive for *Strongyloides stercoralis*. Ivermectin therapy was commenced and continued for a total of 4 weeks. He was discharged for rehabilitation after 25 days.

He had no exposure to endemic countries or to immigrants. During many years he had multiple gastrointestinal symptoms, respiratory symptoms, cutaneous symptoms, chronic eosinophilia, and high immunoglobulin E levels. He underwent several operative procedures and numerous hospitalizations and medical encounters with different experts but a parasitic infection was not considered. His asymptomatic daughter was also found to be serologically positive.

**Conclusions:**

*Strongyloides stercoralis* hyperinfection associated with *Streptococcus pyogenes* bacteremia and toxic shock is described for the first time. The case also highlights the importance of history taking and reviewing past laboratory results, the utility of serological tests for *Strongyloides stercoralis*, and the importance of screening asymptomatic family members of an infected patient. *Strongyloides stercoralis* hyperinfection must be considered in the differential diagnosis of any patient with *Streptococcus pyogenes* bacteremia or toxic shock of no clear source as well as in symptomatic patients with chronic or intermittent eosinophilia, even without any epidemiological risk factors.

## Background

*Strongyloides stercoralis* hyperinfection may be complicated with bloodstream infection of bacteria originating from the intestinal tract or the skin. The bacteria that are typically recovered are Enterobacteriaceae, enteric Gram-positive bacteria such as *Streptococcus bovis* and *Enterococcus faecalis* and typical skin colonizers such as *Staphylococcus aureus* and coagulase-negative staphylococci [1]. We describe a case report of a patient with *Streptococcus pyogenes* (group A streptococcus; GAS) bacteremia and streptococcal toxic shock syndrome (STSS) associated with *S. stercoralis* hyperinfection. To the best of our knowledge these associations have not been reported previously.

## Case presentation

A 78-year-old Israeli man presented to our intensive care unit with fever, flaccid limb weakness, and dysarthria. On the morning of his admission he felt cold and weak. He awoke suddenly with vomiting, weakness of four limbs, and slurred speech. In the emergency room he had a fever of 39.2 °C, blood pressure of 166/118 mmHg, and heart rate of 91 beats per minute. Laboratory tests showed leukocytosis, mild thrombocytopenia, mild eosinophilia (700 cells per microliter), hyponatremia, acute kidney injury, hyperbilirubinemia, elevated liver enzymes (both hepatocellular and cholestatic enzymes), elevated international normalized ratio (INR), metabolic acidosis, and hyperlactatemia. More laboratory results are shown in Table 1. Whole body computed tomography (CT) and CT angiography showed atherosclerosis of the carotid arteries and severe degenerative vertebral discopathy, with no signs of cerebral ischemia and no evidence of spinal epidural abscess. There were small bilateral pleural effusions, ascites, and anasarca. A quick review of his past laboratory results showed long-lasting chronic eosinophilia (reaching 3000 cells/microliter a month before admission) and immunoglobulin E (IgE) level of 1600 kU/L (normal < 214 kU/L) 6 years before admission.

**Table 1:** Laboratory data

Parameter	Normal range	On admission 25 October	HD 2 27 October	HD 4 29 October	HD6 31 October
Hemoglobin (gr%)	13–18	14.7	**11.0**	13.8	12.7
WBC (cells/mcl)	4000–11,000	**17,400**	**13,700**	**16,700**	10,100
PMN (cells/mcl)	2000–8000	**13,000**	**12,100**	**13,600**	7400
Lymphocytes (cells/mcl)	1500–4000	2100	**900**	1500	**1300**
Eosinophils (cells/mcl)	0–400	700	0	100	0
Platelets (cells/mcl)	150,000–400,000	**114,000**	**49,000**	**45,000**	**87,000**
Glucose (mg%)	82–115	**142**	**267**	**36**	**141**
Sodium (mmol/L)	136–145	**130**	139	**133**	145
Total bilirubin (mg%)	0.2–1.2	**2.56**	**1.26**	**3**	2.5
Direct bilirubin (mg%)	0–0.4	**1.1**	–	**1.3**	**1.25**
AST (IU/L)	0–32	**385**	**4708**	**1276**	**300**
ALT (IU/L)	0–33	**194**	**2754**	**2006**	**807**
GGT (IU/L)	8–61	**244**	**161**	**181**	**116**
Alkaline phosphatase (IU/L)	40–130	**262**	130	**176**	94
LDH (IU/L)	240–480	**865**	**2989**	**907**	**543**
Creatinine (mg%)	0.7–1.2	1.08	1.14	0.93	**1.7**
Albumin (gr%)	3.5–5.2	3.94	**3.3**	**3.17**	**2.3**
Globulin (gr%)	2.8–3.5	2.9	2.4	**4.81**	**4.1**
Ammonia (mcmol/L)	16–60	40.5	**66**	**67**	23
Lactate (mmol/L)	0.7–2.1	7.8	–	–	–
C-reactive protein (mg/L)	0–5	–	–	**39**	**46**

*ALT* alanine aminotransferase, *AST* aspartate aminotransferase, *GGT* gamma glutamyl transferase, *HD* day of hospitalization, *LDH* lactate dehydrogenase, *PMN* polymorphonuclear, *WBC* white blood cells

Boldface numbers denote figures out of the normal range

He was a retired lifeguard, and his past medical history included biologic aortic valve replacement 3 years earlier because of aortic stenosis, paroxysmal atrial fibrillation treated with apixaban anticoagulant therapy, an episode of atrial flutter treated with ablation, status post cerebrovascular accident with mild right hemiparesis, coronary artery heart disease and bypass grafting, congestive heart failure, well-controlled type 2 diabetes mellitus, peripheral vascular disease, arterial hypertension, bilateral knee replacement due to osteoarthrosis, spinal stenosis and chronic back and joint pain with recurrent corticosteroid local injections, benign prostatic hypertrophy, and asthma. A month earlier he underwent an inguinal hernia repair and 3 weeks prior to admission he received an inactivated influenza vaccine. He was born in Iraq and immigrated to Israel when he was 12-years old. He recalled only one international travel to Europe several years prior to admission.

Several possibilities were considered for this patient with fever and limb weakness. With regards to infectious diseases they included subacute prosthetic bacterial endocarditis with an embolic stroke, an infectious encephalitis (including herpes viruses, West Nile virus, sandfly encephalitis, *Listeria monocytogenes* rhombencephalitis), atypical bacterial infection (for example, *Mycoplasma*, *Rickettsia*), spinal epidural abscess, and non-infectious conditions such as post infectious/vaccine-related peripheral neuropathy, acute disseminated encephalomyelitis (ADEM), systemic vasculitis including eosinophilic granulomatosis with polyangiitis (Churg–Strauss syndrome), and an autoimmune state such as catastrophic anti-phospholipid syndrome. Blood cultures were taken and antibiotic treatment with ceftriaxone, ampicillin, vancomycin, acyclovir, and doxycycline was commenced. Our patient was admitted to an internal medicine ward and on the following day the neurologic signs resolved. On examination he was coherent, without dysarthria and he had only mild limb weakness. He was dyspneic and complained of abdominal pain. Liver and kidney functions as well as lactate levels, worsened. Repeated CT angiography of his abdomen showed no signs of mesenteric ischemia. He was transferred to the intensive care unit. A lumbar puncture was postponed because of worsening coagulopathy. Blood cultures (two out of four) were positive for *S. pyogenes.* Antibiotic treatment was switched to penicillin G, clindamycin, and intravenous immunoglobulins (IVIG) for presumed STSS. Repeated physical examinations failed to identify the source of the bacteremia. After 3 days his clinical state deteriorated. He complained of severe abdominal pain and profuse diarrhea, the confusion recurred, as well as dyspnea and restlessness. He had non-oliguric renal dysfunction. His Sequential Organ Failure Assessment (SOFA) score increased to 11 and he underwent mechanical ventilation. He received noradrenaline to maintain blood pressure (0.1 mcg/kg per minute). An electroencephalogram (EEG) showed triphasic waves compatible with encephalopathy. During the next few days the clinical and laboratory findings improved gradually, and he was extubated. Transesophageal echocardiography (TEE) showed no vegetations and antibiotic treatment was discontinued after 14 days. On day 12 of hospitalization the serology results from an enzyme-linked immunosorbent assay (ELISA; Scimedx corporation, Denville, NJ, USA) for *S. stercoralis* that was sent on admission, came back positive from the Israeli reference laboratory. A microscopic stool examination showed numerous motile larvae; stool real-time polymerase chain reaction (PCR) for *S. stercoralis* was positive. All other diagnostic tests were negative (Table 2). Our patient was treated with ivermectin (200 mcg/kg). Urine was positive for larvae 8 days after treatment commencement. The treatment was continued for 2 weeks after repeated stool tests became negative; a total of 4 weeks of treatment. No side effects were noted and he was discharged for rehabilitation after 25 days of hospitalization. Repeated stool PCR for *S. stercoralis* after 10 weeks remained negative.

**Table 2:** Diagnostic workup data

Parameter	Results
Blood cultures	2/4 positive for *Streptococcus pyogenes*
Serology tests	
Anti-HAV IgM	Negative
Anti-HCV	Negative
Anti-HBs	239 mIU/ml
HBsAg	Negative
CMV IgG/IgM	Positive/Negative
EBV IgG/IgM/EBNA	Positive/Negative/Positive
*Coxiella burnetii*	Negative
*Rickettsia typhi*	Negative
*Rickettsia conorii*	Negative
Sandfly virus IFA – IgM/IgG	Negative /Negative
*Strongyloides stercoralis* – ELISA	Positive
HTLV 1&2	Negative
Autoimmune panel	
Mitochondrial antibody	Negative
Cardiolipin IgG/IgA	Negative/Negative
Antinuclear antibodies	Borderline
P-ANCA/C-ANCA	Negative
Parasitology tests	
Stool for parasites	Larvae seen
Sputum for parasites	Negative
Urine for parasites	Larvae seen
Stool RT-PCR for *Strongyloides stercoralis*	Positive

*anti-HBs* hepatitis B surface antibody, *C-ANCA* cytoplasmic anti-neutrophil cytoplasmic antibody, *HAV* hepatitis A virus, *HBsAg* hepatitis B surface antigen, *HCV* hepatitis C virus, *CMV* cytomegalovirus, *EBNA* EBV nuclear antigen, *EBV* Epstein–Barr virus, *ELISA* enzyme-linked immunosorbent assay, *HTLV* human T-lymphotropic virus, *IFA* immunofluorescence assay, *Ig* immunoglobulin, *P-ANCA* perinuclear anti-neutrophil cytoplasmic antibody, *PCR* polymerase chain reaction, *RT* real time

## Discussion and conclusions

We present a case of *S. stercoralis* hyperinfection associated with GAS bacteremia. This case is unusual in several aspects: (1) the association between GAS bacteremia and *S. stercoralis* hyperinfection has not been described previously, (2) the association between the presentation as severe multi-organ failure compatible with GAS toxic shock syndrome and *S. stercoralis* hyperinfection, (3) the progression to hyperinfection after seemingly low intensity of immunosuppression related to local corticosteroid injections, and (4) our patient had no exposure to endemic areas in his past. His daughter was infected as well and also denied travel to endemic areas.

The intestinal nematode *S. stercoralis* may cause a chronic, auto-infective cycle with asymptomatic infestation that can last for many years. It may also cause persistent gastrointestinal, dermatologic, and pulmonary symptoms including recurrent asthma with chronic eosinophilia [1]. During the transition to the non-disseminated hyperinfection state, there is an increase in the number and migration rate of the larvae through the gut mucosa, with symptoms still confined to the gastrointestinal and pulmonary tracts. The hyperinfection state may also be accompanied by dissemination of the larvae to compartments other than the typical life cycle sites of auto-infection, such as the central nervous system, the liver, and virtually any other organ. During the process of increased migration, enteric bacteria frequently disseminate to extra-intestinal sterile sites through compromised gut mucosa or by virtue of being carried on the larvae’s surface or within the larval intestinal tract. Bacterial sepsis is a frequent complication. The bacteria that are typically recovered from the blood are Enterobacteriaceae, enteric Gram-positive bacteria (such as *S. bovis* and *E. faecalis*), enteric anaerobes, but also non-fermenters and typical skin colonizers such as *S. aureus* and coagulase-negative staphylococci [1]. A search of PubMed revealed no previously described cases of GAS infection complicating *S. stercoralis* hyperinfection. Given this patient’s long-standing history of perianal disease, we hypothesize that translocation of GAS to the bloodstream occurred through the anal mucosa or perianal skin, which are typical sites of GAS colonization.

The clinical presentation of our case was fever with severe weakness, dysarthria, and quadriparesis that resolved within hours, and could not be explained by a single neurologic focus of ischemia. Unfortunately, a lumbar puncture was not performed and the neurological signs remain unexplained. During the initial days of hospitalization he developed multi-organ failure with hypotension and respiratory failure requiring mechanical ventilation. The condition fulfilled the criteria for STSS (that is, isolation of GAS from blood, hypotension, liver dysfunction, coagulopathy, ascites, and pleural effusions with hypoalbuminemia) [2]. Liver invasion by larvae is associated with granulomatous reaction and cholestatic pattern of liver enzymes disturbance while STSS is associated with a predominantly hepatocellular pattern as in this case, where aspartate aminotransferase (AST) reached levels of 60 times the upper limit of the normal range. Similarly, the severe coagulopathy and pleural or peritoneal fluid accumulation are considered criteria of STSS but have not been frequently described with *S. stercoralis* hyperinfection per se. The simultaneous occurrence of these two rare conditions (STSS and *S. stercoralis* hyperinfection) is exceptional.

*S. stercoralis* hyperinfection typically occurs during a state of immunosuppression, and glucocorticoids are the most frequent culprit [3]. It has been related to high-dose as well as low-dose and even to locally administered steroids [4], as well as without any identified immunosuppression [5, 6]. Our patient received multiple intra-articular corticosteroids injections (the last was given 2 months before admission), and an orally administered high-dose prednisone course due to acute hearing loss 7 months prior to admission. The interval between steroid administration and the occurrence of hyperinfection symptoms has not been clearly defined in the literature but this case shows that steroid exposure even months prior to hyperinfection occurrence may have an impact.

Our patient was born in Iraq but lived in Israel for over 66 years. He had not visited any endemic countries and had no known contact with immigrants. Strongyloidiasis in Israel is mainly an imported disease that is seen mainly in specific high-risk populations, such as Ethiopian Jews [7–10] and possibly among refugees from Africa and other immigrants from endemic countries. There are very few reports of *S. stercoralis* infections from Israel and, curiously enough, during the last 25 years the only report of a native Israeli patient without a clear exposure or a significant stay in an endemic country was also from our facility [11]. A more recent case of strongyloidiasis from Israel was probably acquired in Brazil [12]. The epidemiological picture is further complicated with the fact that our patient’s daughter, who was born in Israel and has not left the country, was also tested (per our request) and found serologically positive for *S. stercoralis*. She was asymptomatic and a fecal examination was negative both microscopically and on PCR for *S. stercoralis*. Other family members including his wife were serologically negative. The source of infection in this family remains a mystery.

**Fig. 1:**
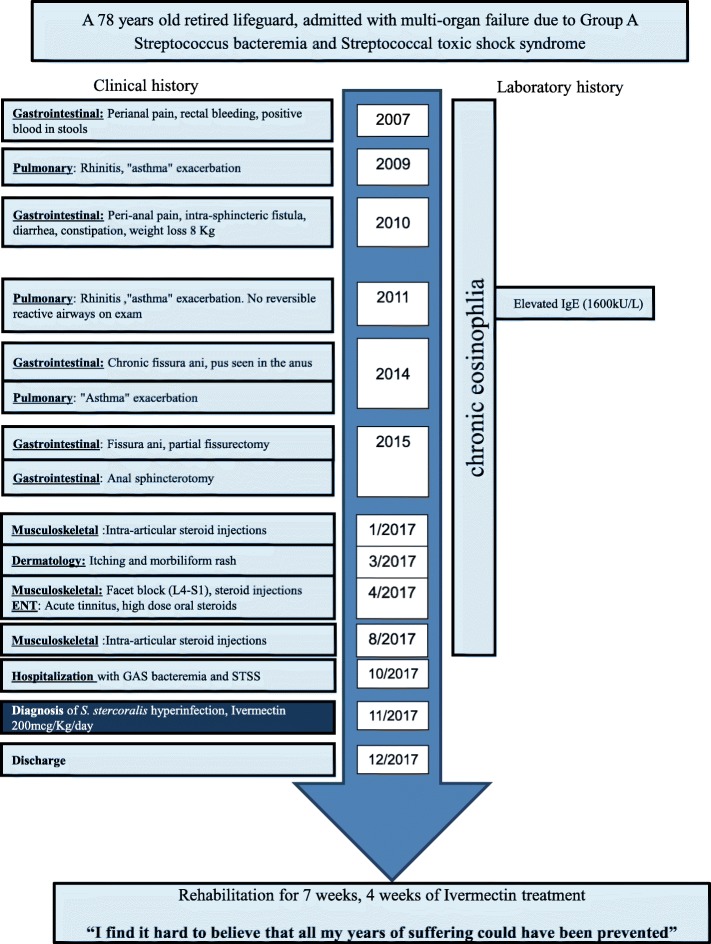
Timeline. *GAS* group A streptococcus, *IgE* immunoglobulin E, *STSS* streptococcal toxic shock syndrome

Our patient reached a state of hyperinfection after years of symptomatic infestation (Timeline, Fig. 1). During many years he suffered from different symptoms including: repeated episodes of rhinitis and “asthma exacerbations” (without signs of reversible reactive airways on directed pulmonology investigations), reported during 2009, 2011, and 2014; rectal bleeding and positive occult blood in stool samples (2007); perianal pain with intra-sphincteric fistula appearance and findings of pus in the anus (2010, 2014); fissura ani and partial fissurectomy (2015); anal sphincterotomy (2015); diarrhea and constipation with weight loss of 8 kg (2010); and the appearance of an itching morbilliform rash (2017). He was hospitalized more than 17 times during the years in several different hospitals and wards and in all cases the eosinophilia was ignored or attributed to asthma. He also had numerous ambulatory encounters with expert physicians including dermatologists, pulmonologists, gastroenterologists, general and cardiac surgeons, cardiologists, and orthopedic surgeons and many sessions with his family physician. He was examined once by an allergy specialist during which he underwent skin patch tests, his IgE levels were obtained, and he was sent once for a stool test for parasites, which was negative. He had documented eosinophilia for at least 13 years, but he had never had serology testing for parasites.

To conclude, we describe for the first time invasive *S. pyogenes* infection and STSS in a patient with *S. stercoralis* hyperinfection. *S. stercoralis* hyperinfection should be considered in any patient with GAS bacteremia of no clear source as well as in patients with chronic or intermittent eosinophilia or elevated IgE levels and in patients with chronic pulmonary, dermatological, and gastrointestinal symptoms, even in the absence of epidemiological risk factors.

## Abbreviations

ADEM: Acute disseminated encephalomyelitis
AST: Aspartate aminotransferase
CT: Computed tomography
EEG: Electroencephalogram
ELISA: Enzyme-linked immunosorbent assay
GAS: Group A streptococcus
IgE: Immunoglobulin E
INR: International normalized ratio
IVIG: Intravenous immunoglobulins
PCR: Polymerase chain reaction
SOFA: Sequential Organ Failure Assessment
STSS: Streptococcal toxic shock syndrome
TEE: Transesophageal echocardiography
